# Psychological Distress, Sleep Quality, and Overall Quality of Life Among Medical Students in a University Mind–Body Exercise Course: An Unlinked Repeated Cross-Sectional Study

**DOI:** 10.3390/healthcare14193196

**Published:** 2026-09-28

**Authors:** Guochun Liu, Jingrong Wang, Shenglan Yan, Pingping Yan, Dongmei Wang

**Affiliations:** 1 College of Exercise Medicine, Chongqing Medical University, Chongqing 400016, China; lgc890206@163.com (G.L.);; 2 Division of Sports Science and Physical Education, Tsinghua University, Beijing 100084, China; 3 Faculty of Education, The Chinese University of Hong Kong, Sha Tin, N.T., Hong Kong 999077, China

**Keywords:** medical students, psychological distress, sleep quality, quality of life, DASS-21, Pittsburgh Sleep Quality Index, Wuqinxi, mind–body exercise

## Abstract

**Background/Objectives:** Psychological distress, poor sleep quality, and reduced quality of life commonly co-occur among medical students, yet their mutually adjusted relationships remain less well characterised within curriculum-based mind–body exercise settings. We examined whether these relationships were replicated across two unlinked survey waves and whether psychological distress and sleep quality remained associated with overall quality of life after mutual adjustment. **Methods:** An unlinked repeated cross-sectional study was embedded within a 12-week university Wuqinxi mind–body exercise course. Anonymous surveys were administered at course initiation (T0) and completion (T1), with 184 valid responses at each wave. Psychological distress, sleep quality, and overall quality of life were assessed using the DASS-21, PSQI, and the PROMIS Global Health Global02 item, respectively. Spearman correlations and proportional-odds ordinal logistic regression were performed with adjustment for sex, age, and previous mind–body exercise experience. Holm correction was applied. **Results:** Associations among the three health domains were directionally consistent at both waves (|ρ| = 0.359–0.494; all Holm-adjusted *p* < 0.001). In mutually adjusted models, poorer sleep quality was associated with lower odds of higher overall quality of life at T0 (OR per SD = 0.375, 95% CI 0.269–0.522) and T1 (OR per SD = 0.400, 95% CI 0.287–0.558; both Holm-adjusted *p* < 0.001). Associations for psychological distress were weaker and did not remain statistically significant after multiplicity correction. **Conclusions:** Across two unlinked survey waves embedded in a 12-week Wuqinxi course, poorer sleep quality remained associated with lower overall quality of life after adjustment for psychological distress and measured covariates, whereas the corresponding association for psychological distress was weaker. These findings characterise concurrent health relationships within the intervention context but do not establish within-person change or causal effects of Wuqinxi.

## 1. Introduction

Medical education spans adolescence and early adulthood, a developmental period characterised by increased vulnerability to mental health problems [1]. Medical training is also associated with high cognitive demands, sustained academic workload, frequent evaluations, and substantial performance expectations [2,3]. Throughout medical education, students encounter additional situational stressors. These include clinical training, patient care responsibilities, exposure to illness and death, and transitions in professional identity [4,5,6]. Even before extensive clinical rotations, early-stage medical students experience substantial stress from intensive coursework, examinations, time constraints, and academic expectations [7,8]. A recent umbrella review incorporating 32 meta-analyses estimated that the pooled prevalence of self-reported psychological and behavioural problems among medical students was approximately 30%, with sleep problems, stress, burnout, anxiety, and depression being particularly prominent [5]. A landmark systematic review published in JAMA further demonstrated that emotional well-being among medical students may deteriorate throughout medical training and highlighted the learning environment itself as an important determinant of student health [3]. Therefore, medical student health research should consider not only psychological symptoms but also potentially modifiable behavioural and recovery-related processes.

Sleep represents an important interface between psychological health, recovery capacity, and academic functioning among medical students. Meta-analytic evidence indicates that poor sleep quality is highly prevalent among medical students (55.6%), with average nightly sleep duration of approximately 6.5 h [9]. Poor sleep quality has also been associated with impaired academic performance [10]. This relationship is likely bidirectional: psychological symptoms may disrupt sleep, whereas persistent sleep impairment may further exacerbate emotional symptoms and daytime dysfunction. Randomised controlled trials additionally suggest that improving sleep may yield benefits for psychological health [11]. Within the high cognitive-load and limited-recovery context of medical education, sleep should not be regarded merely as a secondary symptom of psychological distress. Overall quality of life provides a broader perspective on health beyond symptom burden, with previous studies demonstrating strong associations between anxiety, depression, comorbidity, and poorer quality of life [12]. Because psychological distress and sleep problems are closely related, examining them simultaneously in relation to overall quality of life may clarify their respective conditional associations. Mindfulness and related mind–body approaches have increasingly been incorporated into medical education, with systematic reviews suggesting potential benefits for stress and psychological well-being but substantial heterogeneity across interventions and outcomes [13,14]. Curriculum-based mind–body exercise therefore provides a relevant setting in which to examine how psychological distress, sleep quality, and quality of life coexist, without presuming an effect of the course itself.

The course examined in this study used Wuqinxi, a traditional Chinese mind–body exercise within the qigong tradition [15]. Wuqinxi combines coordinated movement and postural transitions with regulated breathing and attentional engagement, features consistent with the broader construct of meditative movement [15,16]. Evidence from qigong and traditional Chinese exercise studies suggests potential psychological and sleep-related benefits, although intervention heterogeneity and methodological limitations preclude strong conclusions about Wuqinxi-specific effects [17,18]. However, most mechanistic interpretations are extrapolated from broader mindfulness, breathing, and tai chi/qigong research rather than from Wuqinxi-specific evidence [19,20,21].

Although psychological symptoms, sleep disturbance, and quality of life have been examined jointly in medical students [22], intervention studies in medical education have more commonly treated these domains as separate outcomes. Systematic reviews of medical-student interventions have primarily focused on psychological distress, anxiety, depression, stress, and psychological well-being [14,23], whereas sleep-focused reviews have examined sleep quality, insomnia symptoms, and related sleep outcomes [24]. A recent study involving 7128 Chinese university students examined quality of life, stress, sleep anxiety, and sleep quality within an integrated analytical framework, suggesting complex interrelationships among these health domains [25]. However, that study was not specific to medical students and did not directly evaluate the mutually adjusted associations of psychological distress and sleep quality with overall quality of life.

Accordingly, this study examined the relationships of psychological distress and sleep quality with overall quality of life among second-year medical students enrolled in a university mind–body exercise course. We assessed whether these associations remained after mutual adjustment and showed similar patterns across two unlinked survey waves. We hypothesised that greater psychological distress and poorer sleep quality would be associated with lower overall quality of life; the unlinked design and absence of a concurrent control group precluded inference on within-person change or course effects.

## 2. Materials and Methods

### 2.1. Study Design and Estimand

This study used an unlinked repeated cross-sectional design embedded within an existing university physical education course centred on Wuqinxi, a traditional mind–body exercise, at Chongqing Medical University. Anonymous questionnaire surveys were administered at course initiation (T0, 9–13 March 2026) and course completion (T1, 7–13 June 2026). Because no stable identifiers were collected and no concurrent control group was included, individual-level linkage and causal estimation were not possible. Therefore, the primary estimand was the concurrent association structure among psychological distress, sleep quality, and overall quality of life at each survey time point and the consistency of these associations across waves, rather than within-person changes or causal effects attributable to Wuqinxi participation. The study was reported according to the Strengthening the Reporting of Observational Studies in Epidemiology (STROBE) guidelines [26], and the course context was described according to relevant items from the Template for Intervention Description and Replication (TIDieR) checklist [27].

### 2.2. Participants and Course Context

Participants were second-year undergraduate students enrolled in five Wuqinxi course classes through the university’s routine course registration system. The sampling frame comprised all 200 enrolled students (40 per class), all of whom were invited to participate in the research survey at each wave. Instructors introduced the study during class as part of the evaluation of an ongoing physical-activity curriculum reform. Research participation was voluntary, anonymous, and independent of course assessment; participation or non-participation had no effect on course grades. Written informed consent was obtained before participation, and consent was reconfirmed electronically as the first questionnaire item. The anonymous questionnaires were administered electronically using Wenjuanxing (Changsha Ranxing Information Technology Co., Ltd., Changsha, China).

At each survey wave, 184 of the 200 invited students provided valid, consented responses, corresponding to a valid-response rate of 92.0%. Because the surveys were anonymous and no stable identifiers were collected, individual respondents could not be linked across waves and the extent of participant overlap between T0 and T1 cannot be determined. Analytical samples were defined separately by the available valid records at each wave. Because the entire eligible cohort was invited, recruitment was not based on a prospective sample-size target; precision was assessed from the 95% confidence intervals around the primary estimates. All participants were second-year students; participants were drawn from 14 academic schools, with detailed school composition reported in Supplementary Table S1.

The Wuqinxi course was delivered over 12 weeks and comprised one 80 min instructor-led session and one additional in-person group practice session with attendance check-in per week. The instructor-led sessions followed a standardised curriculum delivered by three instructors. Teaching progressed from orientation and foundational movement skills to sequential practice of the five Wuqinxi animal forms, followed by complete-routine consolidation and assessment preparation. Course participation was further supported by video-based review and instructor feedback. Course assessment comprised online platform tasks (30%) and in-person learning and skill performance (70%), together with an end-of-course group competition. The weekly teaching progression, supporting practice, and assessment structure are summarised in Figure 1. The study was approved by the Ethics Committee of Chongqing Medical University (approval No. 2026-128). All participants provided written informed consent before study participation and reconfirmed consent electronically before accessing the online questionnaire.

### 2.3. Measures

#### 2.3.1. Psychological Distress

Psychological distress was assessed using the 21-item Depression Anxiety Stress Scales (DASS-21) [28,29,30,31]. Item responses are conventionally scored from 0 to 3; because the questionnaire platform exported response-position codes from 1 to 4, all item responses were recoded by subtracting 1 before analysis. The DASS-21 comprises depression, anxiety, and stress subscales, while psychometric evidence also supports substantial shared variance and a general distress factor across its items [32]. Because the primary analysis focused on broad psychological distress, the 21 recoded items were summed to yield a total score ranging from 0 to 63, with higher scores indicating greater distress. The total score was not multiplied by two and was not used for clinical diagnosis or case classification. The conventional depression, anxiety, and stress subscales were examined in sensitivity analyses.

#### 2.3.2. Sleep Quality

Sleep quality during the previous month was assessed using the Pittsburgh Sleep Quality Index (PSQI) [33,34]. Following the standard scoring procedure, seven components were calculated: subjective sleep quality, sleep latency, sleep duration, habitual sleep efficiency, sleep disturbances, use of sleep medication, and daytime dysfunction. Each component was scored from 0 to 3, generating a global score ranging from 0 to 21, with higher scores indicating poorer sleep quality.

Because bedtime, wake time, sleep latency, and actual sleep duration included free-text or mixed-format responses, a prespecified rule-based parsing procedure was applied before analysis. Explicit numerical and unambiguous time expressions were converted according to predefined rules. Sleep latency intervals were retained only when the entire interval corresponded to the same PSQI scoring category; intervals crossing scoring thresholds were coded as missing. Sleep-duration intervals, invalid minute values, ambiguous AM/PM expressions, and non-quantifiable text responses were treated as missing in the primary analysis.

Records in which reported sleep duration exceeded calculated time in bed were flagged and examined in corresponding exclusion analyses to assess robustness. PSQI global scores were calculated only when all seven components were available; missing components were not imputed. Detailed PSQI parsing procedures and data quality rules are provided in Supplementary Methods. PSQI completeness, data quality indicators, and component distributions are presented in Supplementary Table S2. Internal consistency across the seven PSQI component scores was summarised descriptively using Cronbach’s *α* among complete cases.

#### 2.3.3. Overall Perceived Quality of Life

Overall perceived quality of life was assessed using the Global02 item from the PROMIS Global Health measure [35,36]. The item asks respondents to rate their overall quality of life from “Excellent” to “Poor”. It was included as a brief global indicator within the broader survey battery and was analysed as a five-category ordinal measure after reverse coding, with higher categories indicating better perceived quality of life. No PROMIS Global Physical Health or Global Mental Health summary score was calculated, and the Global02 item was not interpreted as a multidimensional quality-of-life measure.

#### 2.3.4. Covariates

Adjusted models included sex, age, and previous mind–body exercise experience. These variables were selected before estimation of the core association models on substantive grounds as background characteristics plausibly related to psychological distress, sleep quality, and perceived quality of life, and were consistently available at both survey waves. The covariate set was finalised after data collection but before model fitting; neither the study nor the analytical plan was prospectively registered. Adjustment was therefore intended to account for measured background characteristics rather than to provide comprehensive control of confounding. Academic school was examined only in sensitivity analyses because several categories contained sparse observations. Other potentially relevant factors, including academic workload, physical activity, health status, medication use, chronotype, and lifestyle behaviours, were unavailable and could not be incorporated into the models.

### 2.4. Data Quality, Missing Data, and Analytical Samples

Before analysis, the original datasets were checked for file hashes, dimensions, variable mappings, theoretical value ranges, and duplicate or highly similar response patterns. Similar response vectors were not considered evidence of duplicate participants and therefore were not removed in the primary analysis. Primary analyses used complete records required for each specific model, without statistical imputation. DASS-21 and overall quality of life data contained no missing values in either wave. Valid PSQI sample sizes were 177 at T0 and 180 at T1.

A strict data-quality sensitivity analysis required both criteria to be met: (1) questionnaire completion time below the fifth percentile within the corresponding wave; and (2) at least two of the three multi-item matrices (DASS-21, Brief MAIA-2, and ERQ) showing identical responses across all items. According to the prespecified rules, 5 and 3 records were flagged at T0 and T1, respectively. Data-quality indicators and PSQI completeness are presented in Supplementary Table S2.

### 2.5. Statistical Analysis

Continuous variables were summarised using means and standard deviations and medians and interquartile ranges, whereas categorical variables were summarised using frequencies and percentages. Differences in sample composition between waves were described using standardised differences and were not interpreted as evidence of individual-level change. First, Spearman rank correlation coefficients between DASS-21 total scores, Pittsburgh Sleep Quality Index global score, and overall quality of life were estimated separately at T0 and T1. Ninety-five percent confidence intervals were obtained using percentile bootstrap procedures with 10,000 record-level resamples. The six primary correlations were treated as a single multiplicity family, and the Holm method was applied to control the family-wise error rate.

Primary adjusted analyses modelled overall quality of life as an ordinal outcome using proportional odds ordinal logistic regression separately for each wave. DASS-21 and PSQI scores were entered simultaneously, with adjustment for sex, age, and previous mind–body exercise experience. Both raw-scale and standardised odds ratios (ORs) with 95% confidence intervals were reported. Standardised ORs were calculated using predictor standard deviations from the corresponding complete-case model sample at each wave. The four core predictor estimates (two predictors × two survey waves) were treated as a single multiplicity family, and Holm correction was applied. OR values below 1 indicate that higher predictor levels were associated with lower odds of being classified into higher overall quality of life categories.

The proportional odds assumption was evaluated using Brant-type Wald tests, including both global model tests and predictor-specific tests. These tests were based on binary logistic regression models across cumulative outcome thresholds while accounting for covariance among coefficient estimates from different thresholds within the same model [37]. Sensitivity analyses included Pearson correlations, alternative PSQI upper- and lower-bound parsing schemes, exclusion of strict quality-control flagged records, exclusion of records with inconsistent sleep-efficiency calculations, additional adjustment for academic school, and separate wave-specific ordinal models replacing the DASS-21 total score with the conventional depression, anxiety, and stress subscale scores. The school-adjusted model was considered a sensitivity analysis rather than part of the primary analytical framework. Secondary analyses modelled DASS-21 total scores as a continuous outcome using ordinary least squares regression, with PSQI, overall quality of life, and the same covariates included. HC3 heteroscedasticity-consistent standard errors were applied.

All tests were two-sided. Effect estimates, 95% confidence intervals, and exact *p* values were reported. Interpretation prioritised effect magnitude, precision, and consistency across analytical specifications. Wave-specific descriptive statistics were reported to characterise marginal distributions at each survey time point. No formal between-wave comparison of means was performed. All statistical analyses and verification procedures were conducted using Python 3.13.5 (Python Software Foundation, Wilmington, DE, USA), with pandas 2.2.3, NumPy 2.3.5, SciPy 1.17.0, and statsmodels 0.14.6.

## 3. Results

### 3.1. Sample Characteristics and Data Completeness

Of the 200 students invited at each survey wave, 184 provided valid, consented responses, yielding a valid-response rate of 92.0% at both T0 and T1. All respondents were second-year undergraduate students. Sex distribution was identical and prior mind–body exercise experience was comparable between waves. Among the characteristics presented in Table 1, age showed the largest absolute standardised mean difference (|SMD| = 0.23). DASS-21 total scores and overall quality of life scores had no missing data at either time point, whereas missing values in PSQI global scores were minimal (Table 2). According to the prespecified strict quality-control criteria, 5 and 3 records were flagged at T0 and T1, respectively, and all were retained in the primary analyses. DASS-21 total scores demonstrated high internal consistency at both waves (T0: Cronbach’s *α* = 0.948, McDonald’s *ω* = 0.951; T1: *α* = 0.957, *ω* = 0.960). Cronbach’s *α* across the seven PSQI component scores was 0.630 at T0 and 0.697 at T1 (Supplementary Table S3). Detailed PSQI parsing results and data-quality indicators are presented in Supplementary Table S2.

### 3.2. Distribution of Core Study Variables

The mean (SD) DASS-21 total scores were 10.45 (9.89) at T0 and 8.47 (9.21) at T1. The corresponding PSQI global scores were 4.38 (2.41) and 4.66 (2.60), respectively. Mean overall quality of life scores were 3.60 (0.94) at T0 and 3.70 (1.00) at T1 (Table 2). These statistics describe wave-specific marginal distributions and do not represent within-person or course-related change.

### 3.3. Concurrent Associations Among Psychological Distress, Sleep Quality, and Overall Quality of Life

The three core variables demonstrated directionally consistent concurrent associations across the two survey time points, with the absolute values of the six Spearman correlation coefficients ranging from 0.359 to 0.494 (Table 3).

At T0, DASS-21 scores were positively correlated with PSQI global scores (ρ = 0.392, 95% bootstrap CI 0.251–0.523). DASS-21 scores were negatively correlated with overall quality of life (ρ = −0.398, 95% bootstrap CI −0.522 to −0.262), and PSQI global scores also showed a negative correlation with overall quality of life (ρ = −0.460, 95% bootstrap CI −0.584 to −0.327). At T1, the corresponding correlation coefficients were 0.447 (95% bootstrap CI 0.314–0.563), −0.359 (95% bootstrap CI −0.480 to −0.227), and −0.494 (95% bootstrap CI −0.603 to −0.368), respectively. After applying Holm correction with the six core correlations treated as a single multiple-testing family, all adjusted *p* values remained <0.001. These findings indicate that the concurrent associations among higher psychological distress, poorer sleep quality, and lower overall quality of life were directionally consistent across the two survey time points.

### 3.4. Mutually Adjusted Associations of Psychological Distress and Sleep Quality with Overall Quality of Life

After simultaneously including DASS-21 and PSQI scores and adjusting for sex, age, and prior experience with mind–body exercise, PSQI remained consistently associated with lower odds of being classified into higher overall quality of life categories at both survey time points (Table 4). After standardisation based on predictor-specific standard deviations within the corresponding complete-case model samples, each 1-SD increase in PSQI was associated with lower odds of higher overall quality-of-life levels, with ORs of 0.375 (95% CI, 0.269–0.522) at T0 and 0.400 (95% CI, 0.287–0.558) at T1 (both Holm-adjusted *p* < 0.001). The mutually adjusted associations for DASS-21 were weaker, with corresponding ORs of 0.733 (95% CI, 0.536–1.002) at T0 and 0.709 (95% CI, 0.524–0.959) at T1. After applying Holm correction to the four prespecified core coefficients, the adjusted *p* values were 0.052 and 0.051, respectively. Brant-type global tests provided no evidence of violation of the proportional odds assumption at either T0 [*χ*^2^(10) = 9.33, *p* = 0.501] or T1 [*χ*^2^(10) = 13.81, *p* = 0.182]. Predictor-specific tests for both DASS-21 and PSQI were also non-significant (*p* > 0.05; Supplementary Table S4).

### 3.5. Sensitivity and Secondary Analyses

The primary correlation patterns and the mutually adjusted association between PSQI and overall quality of life remained broadly consistent across alternative analytical specifications. Pearson and Spearman correlations showed concordant directions and did not alter the primary association patterns. The core correlation findings were also unchanged after excluding strict quality-control flagged records, applying alternative upper- and lower-bound PSQI parsing schemes, and excluding records with inconsistent sleep-efficiency calculations (Supplementary Table S5).

In ordinal logistic sensitivity analyses, standardised ORs for PSQI ranged from 0.351 to 0.420 across data-quality, parsing, and covariate specifications (Supplementary Table S6). Replacing the DASS-21 total score in turn with the depression, anxiety, or stress subscale similarly yielded stable PSQI estimates (OR per SD, 0.366–0.407), whereas the distress-subscale estimates were consistently inverse but varied in precision; the T0 anxiety and stress confidence intervals included 1 (Supplementary Table S7). DASS-21 total-score estimates were also more sensitive to covariate specification: after additional adjustment for academic school, the ORs were 0.738 (95% CI, 0.528–1.030) at T0 and 0.751 (95% CI, 0.541–1.043) at T1 (Supplementary Table S6).

In secondary HC3 robust linear regression models, each 1-point increase in PSQI was associated with a 1.13-point higher DASS-21 score at T0 (95% CI, 0.40–1.85) and a 1.06-point higher score at T1 (95% CI, 0.49–1.63). Overall quality of life was inversely associated with DASS-21 scores at both waves, with coefficients of −1.57 (95% CI, −3.23 to 0.09) at T0 and −1.87 (95% CI, −3.38 to −0.37) at T1 (Supplementary Table S8). These secondary models represent concurrent conditional associations and do not establish temporal or causal relationships.

## 4. Discussion

### 4.1. Main Findings

Across two unlinked course-anchored surveys, greater psychological distress, poorer sleep quality, and lower overall quality of life showed consistent within-wave associations. When DASS-21 and PSQI scores were modelled jointly, poorer sleep quality remained associated with lower odds of higher overall quality of life at both waves, whereas DASS-21 estimates were attenuated and more sensitive to model specification. The principal contribution is therefore the replication of this conditional association pattern across two cross-sectional samples rather than evidence of temporal stability or construct independence.

### 4.2. Sleep, Psychological Distress, and Overall Quality of Life

Our findings are consistent with the broader evidence indicating that poor sleep is highly prevalent among medical students and is associated with impaired academic functioning and reduced well-being. Large-scale meta-analyses have identified poor sleep quality as one of the most common health concerns among medical students, with sleep disturbances linked to adverse educational outcomes [5,9,10]. The relationship between sleep and psychological health is unlikely to be unidirectional. Longitudinal evidence among healthcare students suggests reciprocal temporal associations between sleep quality and internalizing symptoms, including depression and anxiety [38], while randomised trial meta-analyses indicate that improving sleep may lead to improvements in depression, anxiety, and stress outcomes [11].

After adjustment for psychological distress, PSQI remained associated with overall quality of life at both survey waves, indicating that the observed sleep–quality-of-life association persisted when concurrent psychological distress and measured covariates were considered. Recent evidence among Chinese university students similarly indicates interrelationships among quality of life, perceived stress, sleep anxiety, and sleep quality [25]. Research on psychological comorbidity among university students has also demonstrated strong associations between symptom burden and poorer quality of life [12]. In medical education, where sustained cognitive and evaluative demands are well recognised, sleep therefore remains a relevant dimension of student health, although the present findings do not establish incremental clinical utility beyond psychological symptom measures.

The attenuation of the DASS-21 association after adjustment for PSQI warrants careful interpretation. Although psychological distress was associated with overall quality of life in unadjusted analyses, its conditional estimate was reduced when sleep quality was included in the model. Given the concurrent cross-sectional design and the recognised bidirectional relationship between sleep and psychological symptoms [38], this attenuation cannot distinguish shared variance, confounding, mediation, or reverse relationships; common-method variance from concurrent self-report measures may also have contributed.

### 4.3. Interpretation Within the Mind–Body Exercise Course Context

The study was conducted within a curriculum-based mind–body exercise course centred on Wuqinxi. Because all participants were enrolled in the same course and the two surveys were unlinked, the present design does not estimate within-person change or course effects. The broadly similar association patterns observed at T0, before substantial course exposure, and at T1, after course completion, do not support attributing these relationships to Wuqinxi.

Evidence from intervention studies provides relevant context but does not alter this interpretation. Meta-analyses of randomised trials in university students suggest that qigong and related traditional Chinese exercises may improve depressive and anxiety symptoms and sleep outcomes, although the underlying evidence is limited by heterogeneity and methodological weaknesses [17,18]. For sleep specifically, a meta-analysis of Health Qigong trials reported substantial heterogeneity and possible nonspecific intervention effects [39], while a recent network meta-analysis that included Wuqinxi was conducted in patients with insomnia [40]. These findings therefore cannot be directly extrapolated to medical students enrolled in a routine physical-education course. Accordingly, the present findings characterise the association structure observed within a curriculum-based mind–body exercise context; they do not establish that Wuqinxi altered these outcomes or their interrelationships.

### 4.4. Implications for Research and Medical Education

Medical student well-being is shaped by educational, social, behavioural, and psychological influences, including the learning environment, workload, assessment demands, and individual health behaviours [3,7]. The present findings support considering sleep alongside psychological symptoms when characterising student health, but do not establish that sleep assessment provides incremental predictive or clinical utility. Prospective studies are required to determine whether sleep measures improve prediction of subsequent functioning, academic outcomes, or multidimensional well-being beyond psychological symptoms.

At the curriculum level, evidence syntheses indicate that interventions targeting medical student well-being vary substantially in content, delivery, and outcome selection, while the overall certainty of evidence remains limited [41]. Curriculum-embedded mind–body exercise should therefore be evaluated using linked longitudinal or controlled designs with preregistered outcomes and measures of attendance, practice dose, and implementation fidelity. Incorporating sleep, psychological distress, quality of life, and theoretically relevant process measures within the same analytical framework would allow future studies to distinguish changes in health outcomes from potential mechanisms of course exposure.

### 4.5. Limitations

Several limitations should be considered. First, the two survey waves could not be linked at the individual level, the degree of respondent overlap is unknown, and no concurrent control group was available; consequently, the study cannot estimate within-person change or causal course effects. Second, the sample was drawn from a single medical university, consisted exclusively of second-year students, and included a high proportion of female participants, limiting generalisability and potentially reducing the precision of sex-adjusted estimates. Third, psychological distress, sleep quality, and quality of life were assessed using concurrent self-reported measures, and overall quality of life was evaluated using a single-item measure; therefore, common-method bias and limited domain-specific information remain possible. Fourth, several PSQI components required reconstruction from free-text responses. Although alternative parsing strategies produced similar findings, measurement error cannot be completely excluded. Fifth, available covariates did not account for potential residual confounding from academic workload, previous mental health status, circadian patterns, or lifestyle behaviours. The study was not prospectively registered. In addition, the end-of-course questionnaire introduction mentioned potential health benefits of the course, which may have contributed to expectancy effects or socially desirable responses.

## 5. Conclusions

Across two course-anchored survey time points, psychological distress, sleep quality, and overall quality of life demonstrated directionally consistent concurrent associations among medical students. After mutual adjustment, poorer sleep quality remained associated with lower overall quality-of-life ratings at both waves, whereas associations for psychological distress were weaker and more sensitive to model specification. Because respondents could not be linked and no concurrent control group was available, these findings do not support conclusions regarding within-person change or causal effects of the course. Future longitudinal controlled studies are needed to determine temporal and intervention-related relationships.

## Figures and Tables

**Figure 1:**
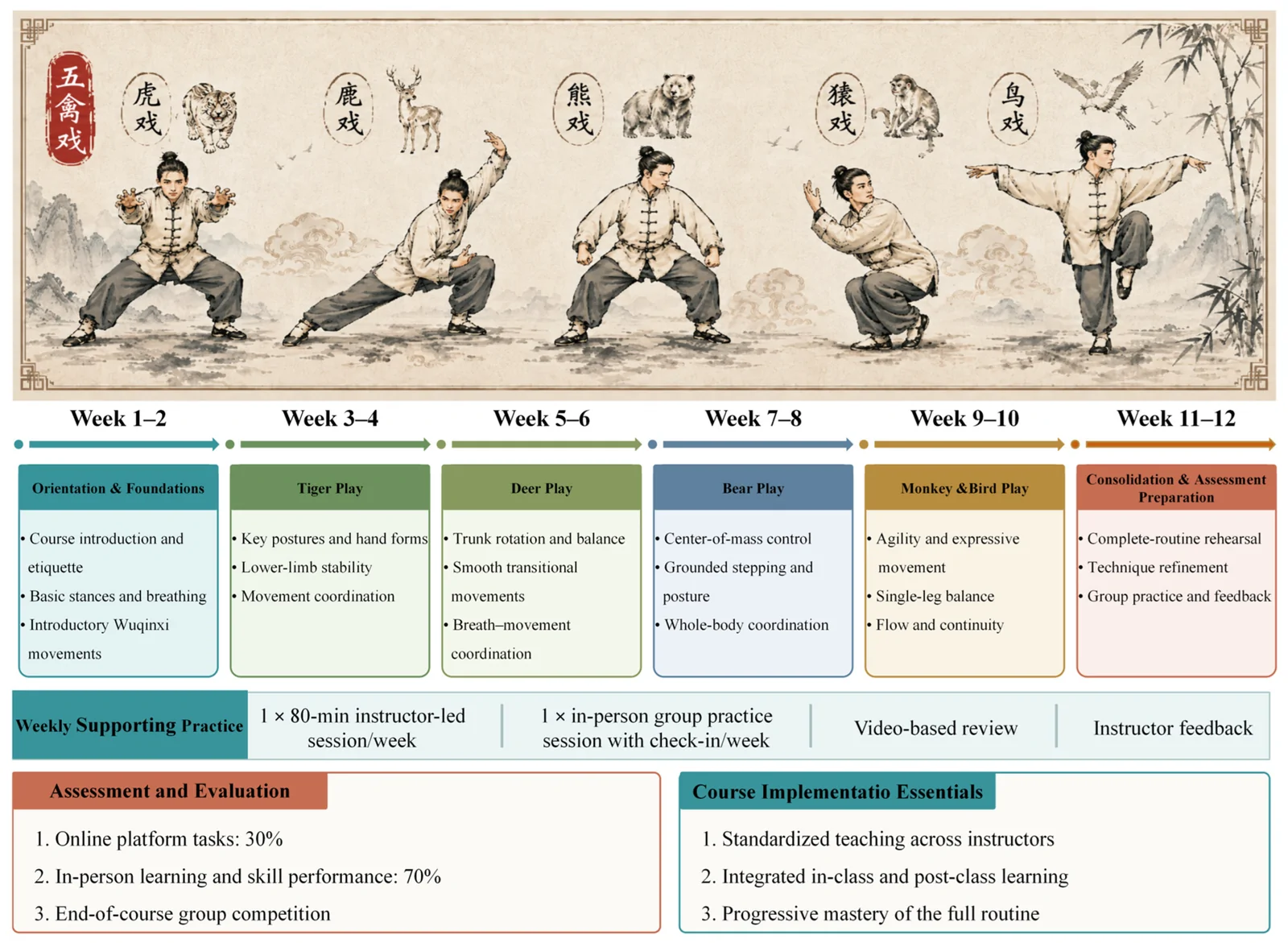
Timeline and implementation structure of the 12-week Wuqinxi course. The Chinese characters in the upper illustration denote Wuqinxi (五禽戏, Five-Animal Exercises) and its five animal forms: tiger (虎戏), deer (鹿戏), bear (熊戏), ape (猿戏), and bird (鸟戏).

**Table 1:** Participant characteristics by survey wave.

Characteristic	T0 (*n* = 184)	T1 (*n* = 184)	|SMD|
Age, years, mean (SD)	19.60 (0.61)	19.73 (0.58)	0.23
Sex, *n* (%)			0.00
Female	156 (84.8)	156 (84.8)	
Male	28 (15.2)	28 (15.2)	
Previous mind–body exercise experience, *n* (%)	131 (71.2)	135 (73.4)	0.05

Note. Values are *n* (%) unless otherwise indicated. SMD, standardised mean difference. Absolute SMDs describe marginal differences in sample composition between the two unlinked survey waves and do not represent within-participant change. All participants were second-year undergraduates.

**Table 2:** Distribution of core study variables at the two survey time points.

Wave	Variable	*n*	Missing, *n*	Mean (SD)	Median (IQR)	Observed Range
T0	DASS-21 total	184	0	10.45 (9.89)	7.00 (2.00–17.00)	0–42
T0	PSQI global	177	7	4.38 (2.41)	4.00 (3.00–6.00)	0–12
T0	Global perceived quality-of-life rating	184	0	3.60 (0.94)	4.00 (3.00–4.00)	2–5
T1	DASS-21 total	184	0	8.47 (9.21)	5.50 (0.00–13.25)	0–42
T1	PSQI global	180	4	4.66 (2.60)	4.00 (3.00–6.00)	0–13
T1	Global perceived quality-of-life rating	184	0	3.70 (1.00)	4.00 (3.00–5.00)	2–5

Note. DASS-21 scores range from 0 to 63 and PSQI global scores from 0 to 21; higher scores indicate greater psychological distress and poorer sleep quality, respectively. Responses were coded from 1 to 5 after reverse ordering, with higher values indicating better quality of life. The two survey waves were unlinked; between-wave differences are descriptive only.

**Table 3:** Within-wave concurrent associations among psychological distress, sleep quality, and overall quality of life.

Wave	Association	*n*	Spearman ρ	95% Bootstrap CI	Raw *p*	Holm-Adjusted *p*
T0	DASS-21 total–PSQI global	177	0.392	0.251–0.523	<0.001	<0.001
T0	DASS-21 total–Overall quality of life	184	−0.398	−0.522 to −0.262	<0.001	<0.001
T0	PSQI global–Overall quality of life	177	−0.460	−0.584 to −0.327	<0.001	<0.001
T1	DASS-21 total–PSQI global	180	0.447	0.314–0.563	<0.001	<0.001
T1	DASS-21 total–Overall quality of life	184	−0.359	−0.480 to −0.227	<0.001	<0.001
T1	PSQI global–Overall quality of life	180	−0.494	−0.603 to −0.368	<0.001	<0.001

Note. CIs are percentile-bootstrap 95% confidence intervals based on 10,000 record-level resamples. Holm adjustment was applied across the six focal correlations. Higher DASS-21 and PSQI scores indicate worse status, whereas higher Overall quality of life ratings indicate better quality of life.

**Table 4:** Mutually adjusted associations of psychological distress and sleep quality with overall quality of life categories.

Wave	Predictor	*n*	OR per Raw Unit (95% CI)	OR per SD (95% CI)	Raw *p*	Holm-Adjusted *p*
T0	DASS-21 total	177	0.968 (0.937–1.000)	0.733 (0.536–1.002)	0.052	0.052
T0	PSQI global	177	0.665 (0.580–0.764)	0.375 (0.269–0.522)	<0.001	<0.001
T1	DASS-21 total	180	0.964 (0.933–0.995)	0.709 (0.524–0.959)	0.026	0.051
T1	PSQI global	180	0.703 (0.619–0.799)	0.400 (0.287–0.558)	<0.001	<0.001

Note. Separate proportional-odds ordinal logistic regression models were fitted at T0 and T1. Each model included DASS-21 and PSQI jointly and adjusted for sex, age, and previous mind–body exercise experience. ORs per SD were calculated using predictor SDs from the corresponding wave-specific complete-case model sample. ORs below 1 indicate lower odds of being in a higher global quality-of-life category. Holm adjustment was applied across the four focal predictor coefficients. Global Brant-type tests showed no evidence of violation of the proportional-odds assumption at T0 (*p* = 0.501) or T1 (*p* = 0.182).

## Data Availability

The de-identified data supporting the findings of this study are not publicly available because they contain participant-level information collected under institutional ethical oversight. De-identified data and the analysis code may be made available by the corresponding author upon reasonable request, subject to approval under applicable institutional and ethical requirements.

## Supplementary Materials

The following supporting information can be downloaded at https://www.mdpi.com/article/10.3390/healthcare14193196/s1. Supplementary Methods: PSQI reconstruction and data-quality rules; Table S1: Full participant characteristics of the two survey waves; Table S2: PSQI reconstruction, completeness, and component distributions; Table S3: Internal consistency of DASS-21 and PSQI scores by survey wave; Table S4: Brant-type tests of the proportional-odds assumption; Table S5: Sensitivity analyses for concurrent correlations; Table S6: Sensitivity analyses for ordinal regression models; Table S7: Sensitivity analyses replacing the DASS-21 total score with conventional subscale scores; Table S8: Secondary HC3 models for DASS-21 total scores.
