# Supplementary material for: Psychological Distress, Sleep Quality, and Overall Quality of Life Among Medical Students in a University Mind–Body Exercise Course: An Unlinked Repeated Cross-Sectional Study
Source: Healthcare (Basel). 2026 Sep 28;14(19):3196. doi: 10.3390/healthcare14193196 (PMC13664894; doi:10.3390/healthcare14193196)
Supplement: Supplementary file 1 [file healthcare-14-03196-s001.zip › healthcare-4561999-supplementary.pdf]

Supplementary Materials

Psychological Distress, Sleep Quality, and Overall Quality of Life among Medical Students in a University Mind－Body Exercise Course: An Unlinked Repeated Cross-Sectional Study

This additional file contains the supplementary analytic methods and tables cited in the main manuscript. The two survey waves were unlinked; all supplementary analyses are interpreted as wave-specific concurrent associations or sensitivity analyses and not as within-person change or causal effects of the course.

Supplementary Methods

PSQI reconstruction and data-quality rules

PSQI components were reconstructed from the questionnaire fields using a frozen rule set before the focal association models were fitted. Clock-time fields were accepted only when they could be interpreted unambiguously. Minute values outside 0–59, ambiguous AM/PM expressions, nonquantifiable text, and sleep-duration ranges were treated as missing in the main analysis. For the evening bedtime prompt, 9–11 were interpreted as 21:00–23:59, 12/24 as 00:00–00:59, and explicit values of 21–23 or 0–4 were retained; other ambiguous values were set to missing. Morning wake times of 5–12 were interpreted directly. Sleep-latency ranges were retained only when the complete range mapped to one PSQI scoring category; ranges crossing a scoring boundary were set to missing. Unambiguous Chinese numeral/time expressions were normalized before scoring.

Habitual sleep efficiency was calculated from reported sleep duration and derived time in bed. Records in which reported sleep duration exceeded time in bed were flagged; efficiency was capped at 100% for component classification, and exclusion of flagged records was examined in sensitivity analyses. A PSQI global score was calculated only when all seven components were available; no component-level imputation was performed. Lower- and upper-bound parser variants were used only as sensitivity analyses for ambiguous but quantifiable entries.

The strict data-quality sensitivity flag required both (1) completion time strictly below the wave-specific fifth percentile and (2) complete straightlining in at least two of the DASS-21, Brief MAIA-2, and ERQ item matrices. Flagged records were retained in the primary analyses and excluded only in sensitivity analyses. Identical or highly similar response vectors were not treated as duplicate participants because stable participant identifiers were unavailable.

Table S1. Full participant characteristics of the two survey waves

variable	category	T0	T1	SMD
Sex	Male	28 (15.2%)	28 (15.2%)	0.000
	Female	156 (84.8%)	156 (84.8%)	0.000
Age, years	18	1 (0.5%)	1 (0.5%)	0.000
	19	83 (45.1%)	59 (32.1%)	-0.270
	20	89 (48.4%)	112 (60.9%)	0.253
	21	11 (6.0%)	12 (6.5%)	0.022
Previous mind–body exercise experience	No	53 (28.8%)	49 (26.6%)	−0.049
	Yes	131 (71.2%)	135 (73.4%)	0.049
College	School of Basic Medicine	3 (1.6%)	2 (1.1%)	-0.047
	School of Laboratory Medicine	8 (4.3%)	10 (5.4%)	0.050
	School of Public Health	9 (4.9%)	9 (4.9%)	0.000
	School of Pharmacy	7 (3.8%)	7 (3.8%)	0.000

variable	category	T0	T1	SMD
	School of Biomedical Engineering	1 (0.5%)	3 (1.6%)	0.105
	Medical English College	4 (2.2%)	4 (2.2%)	0.000
	School of Medical Informatics	2 (1.1%)	1 (0.5%)	-0.060
	School of Nursing	38 (20.7%)	34 (18.5%)	-0.055
	International Medical School	6 (3.3%)	6 (3.3%)	0.000
	School of Pediatrics	4 (2.2%)	4 (2.2%)	0.000
	First Clinical Medical College	71 (38.6%)	70 (38.0%)	-0.011
	Second Clinical Medical College	21 (11.4%)	23 (12.5%)	0.034
	School of Stomatology	2 (1.1%)	2 (1.1%)	0.000
	Fifth Clinical Medical College	8 (4.3%)	9 (4.9%)	0.026

Note. SMD, standardized mean difference. Signed category-specific SMDs are descriptive measures of marginal composition differences; no between-wave hypothesis test was performed. One questionnaire-listed college had no participants in either wave and was omitted from the table.

Table S2. PSQI reconstruction, completeness, and component distributions

Panel A. Data-quality and parsing summary

Wave	n	5th percentile completion time, s	Strict QC flags	DASS straightliners	MAIA straightliners	ERQ straightliners	PSQI global missing	PSQI manual review
T0	184	167	5	27	10	30	7	34
T1	184	135	3	58	9	45	4	20

Panel B. PSQI component completeness and distributions

**Wave**	**PSQI score/component**	**n**	**Missing, n**	**Mean (SD)**	**Median (IQR)**	**Observed range**
T0	Subjective sleep quality	184	0	0.82 (0.63)	1 (0–1)	0–3
T0	Sleep latency	178	6	0.80 (0.83)	1 (0–1)	0–3
T0	Sleep duration	183	1	0.36 (0.55)	0 (0–1)	0–3
T0	Habitual sleep efficiency	183	1	0.20 (0.57)	0 (0–0)	0–3
T0	Sleep disturbance	184	0	0.88 (0.51)	1 (1–1)	0–3
T0	Sleeping medication	184	0	0.02 (0.18)	0 (0–0)	0–2
T0	Daytime dysfunction	184	0	1.40 (0.86)	1 (1–2)	0–3
T0	PSQI global	177	7	4.38 (2.41)	4 (3–6)	0–12

Wave	PSQI score/component	n	Missing, n	Mean (SD)	Median (IQR)	Observed range
T1	Subjective sleep quality	184	0	0.90 (0.65)	1 (0–1)	0–2
T1	Sleep latency	182	2	0.87 (0.77)	1 (0–1)	0–3
T1	Sleep duration	183	1	0.50 (0.53)	0 (0–1)	0–2
T1	Habitual sleep efficiency	182	2	0.22 (0.49)	0 (0–0)	0–2
T1	Sleep disturbance	184	0	0.85 (0.54)	1 (1–1)	0–2
T1	Sleeping medication	184	0	0.09 (0.40)	0 (0–0)	0–3
T1	Daytime dysfunction	184	0	1.24 (0.87)	1 (1–2)	0–3
T1	PSQI global	180	4	4.66 (2.60)	4 (3–6)	0–13

Note. PSQI, Pittsburgh Sleep Quality Index; QC, quality control. Component scores range from 0 to 3. The global score ranges from 0 to 21 and was calculated only when all seven components were available. Manual review counts refer to records requiring review of free-text or mixed-format PSQI fields, not exclusions from the primary analysis.

Table S3. Internal consistency of DASS-21 and PSQI scores by survey wave

Wave	Scale	Items	n	Cronbach α	McDonald ω
T0	DASS-21 total	21	184	0.948	0.951
T0	Depression	7	184	0.841	0.863
T0	Anxiety	7	184	0.841	0.864
T0	Stress	7	184	0.891	0.893
T1	DASS-21 total	21	184	0.957	0.960
T1	Depression	7	184	0.879	0.890
T1	Anxiety	7	184	0.860	0.880
T1	Stress	7	184	0.907	0.909
T0	PSQI component scores	7	177	0.630	—
T1	PSQI component scores	7	180	0.697	—

Note. DASS-21, Depression Anxiety Stress Scales－21; PSQI, Pittsburgh Sleep Quality Index. Omega estimates for DASS-21 are one-factor reliability estimates and are reported as reliability sensitivity analyses rather than evidence of structural validity. PSQI reliability was summarised descriptively using Cronbach's α across the seven component scores.McDonald's ω was not calculated for the PSQI component scores.

Table S4. Brant-type tests of the proportional-odds assumption

Panel A. Global and predictor-specific Brant-type Wald tests

Wave	Term	χ^2	df	P
T0	Global test	9.330	10	0.501
T0	DASS-21 total	0.115	2	0.944
T0	PSQI global	4.741	2	0.093
T0	Female sex	0.654	2	0.721
T0	Age	2.336	2	0.311
T0	Previous mind–body practice	0.429	2	0.807
T1	Global test	13.807	10	0.182
T1	DASS-21 total	3.370	2	0.185
T1	PSQI global	0.672	2	0.714
T1	Female sex	0.301	2	0.860
T1	Age	5.024	2	0.081
T1	Previous mind–body practice	2.552	2	0.279

Panel B. Cutpoint-specific cumulative-logit slopes for focal predictors

Wave	Predictor	QoL > 2, β	QoL > 3, β	QoL > 4, β	Brant-type P
T0	DASS-21 total	−0.032	−0.038	−0.029	0.944
T0	PSQI global	−0.305	−0.528	−0.337	0.093
T1	DASS-21 total	−0.058	−0.026	−0.056	0.185
T1	PSQI global	−0.318	−0.388	−0.331	0.714

Note. The Brant-type Wald test evaluates equality of cumulative-logit slopes across observed global quality-of-life cutpoints while accounting for covariance among cutpoint-specific estimates from the same participants. Neither global test rejected the proportional-odds assumption. Panel B is an audit display for the two focal predictors and is not a separate inferential analysis.

Table S5. Sensitivity analyses for concurrent correlations

Panel A. Pearson correlation sensitivity analysis

Wave	Association	n	Pearson r	95% CI	Raw P
T0	DASS-21 total–PSQI global	177	0.375	0.241–0.495	<0.001
T0	DASS-21 total–Global QoL	184	−0.337	−0.459 to −0.202	<0.001

Wave	Association	n	Pearson r	95% CI	Raw P
T0	PSQI global–Global QoL	177	−0.472	−0.579 to −0.349	<0.001
T1	DASS-21 total–PSQI global	180	0.395	0.264–0.512	<0.001
T1	DASS-21 total–Global QoL	184	−0.345	−0.466 to −0.211	<0.001
T1	PSQI global–Global QoL	180	−0.475	−0.581 to −0.353	<0.001

Panel B. Spearman correlations under data-quality and PSQI parsing variants

Wave	Specification	Association	n	Spearman ρ	Raw P
T0	Strict QC excluded	DASS-21–PSQI	172	0.424	<0.001
T0	Strict QC excluded	DASS-21 total ‑ Global QoL	179	−0.398	<0.001
T0	Strict QC excluded	PSQI–QoL	172	−0.451	<0.001
T0	Sleep-efficiency inconsistency excluded	DASS-21–PSQI	154	0.440	<0.001
T0	Sleep-efficiency inconsistency excluded	PSQI–QoL	154	−0.471	<0.001
T0	PSQI lower-bound parser	DASS-21–PSQI	182	0.409	<0.001
T0	PSQI lower-bound parser	PSQI–QoL	182	−0.479	<0.001
T0	PSQI upper-bound parser	DASS-21–PSQI	182	0.407	<0.001
T0	PSQI upper-bound parser	PSQI–QoL	182	−0.477	<0.001
T1	Strict QC excluded	DASS-21–PSQI	177	0.476	<0.001
T1	Strict QC excluded	DASS-21 total ‑ Global QoL	181	−0.373	<0.001
T1	Strict QC excluded	PSQI–QoL	177	−0.489	<0.001
T1	Sleep-efficiency inconsistency excluded	DASS-21–PSQI	168	0.463	<0.001
T1	Sleep-efficiency inconsistency excluded	PSQI–QoL	168	−0.489	<0.001
T1	PSQI lower-bound parser	DASS-21–PSQI	181	0.447	<0.001
T1	PSQI lower-bound parser	PSQI–QoL	181	−0.497	<0.001
T1	PSQI upper-bound parser	DASS-21–PSQI	181	0.447	<0.001
T1	PSQI upper-bound parser	PSQI–QoL	181	−0.497	<0.001

Note. The Pearson analysis assesses dependence on correlation metric. The remaining analyses assess the effect of strict data-quality exclusions and alternative PSQI parsing decisions. Raw P values are shown for sensitivity analyses; confirmatory multiplicity control is reported for the six focal Spearman correlations in the main manuscript.

Table S6. Sensitivity analyses for ordinal regression models

Wave	Specification	n	Predictor	OR per SD	95% CI	Raw P
T0	Strict QC excluded	172	DASS-21 total	0.725	0.529–0.995	0.047
T0	Strict QC excluded	172	PSQI global	0.394	0.282–0.551	<0.001
T0	Sleep-efficiency inconsistency excluded	154	DASS-21 total	0.676	0.479–0.954	0.026
T0	Sleep-efficiency inconsistency excluded	154	PSQI global	0.369	0.257–0.531	<0.001
T0	PSQI lower-bound parser	182	DASS-21 total	0.715	0.521–0.982	0.038
T0	PSQI lower-bound parser	182	PSQI global	0.351	0.250–0.493	<0.001
T0	PSQI upper-bound parser	182	DASS-21 total	0.710	0.518–0.974	0.034
T0	PSQI upper-bound parser	182	PSQI global	0.356	0.254–0.498	<0.001
T0	College adjusted	177	DASS-21 total	0.738	0.528–1.030	0.074
T0	College adjusted	177	PSQI global	0.382	0.271–0.538	<0.001
T1	Strict QC excluded	177	DASS-21 total	0.673	0.491–0.922	0.014
T1	Strict QC excluded	177	PSQI global	0.420	0.299–0.590	<0.001
T1	Sleep-efficiency inconsistency excluded	168	DASS-21 total	0.715	0.524–0.976	0.035
T1	Sleep-efficiency inconsistency excluded	168	PSQI global	0.400	0.283–0.567	<0.001
T1	PSQI lower-bound parser	181	DASS-21 total	0.710	0.525–0.959	0.026
T1	PSQI lower-bound parser	181	PSQI global	0.396	0.284–0.552	<0.001
T1	PSQI upper-bound parser	181	DASS-21 total	0.709	0.525–0.958	0.025
T1	PSQI upper-bound parser	181	PSQI global	0.396	0.284–0.552	<0.001
T1	College adjusted	180	DASS-21 total	0.751	0.541–1.043	0.088
T1	College adjusted	180	PSQI global	0.365	0.258–0.516	<0.001

Note. Each sensitivity model included DASS-21 and PSQI simultaneously and adjusted for sex, age, and previous systematic mind–body practice, with college added only in the college-adjusted specification. ORs are standardized within the corresponding complete-case analysis sample. P values are descriptive sensitivity results and are not used as an additional confirmatory testing family. Proportional-odds diagnostics for the primary models are reported in Table S4.

Table S7. Sensitivity analyses replacing the DASS-21 total score with conventional subscale scores

Wave	DASS-21 subscale	n	Subscale OR per SD (95% CI)	Raw *P*	PSQI OR per SD (95% CI)	Raw *P*
T0	Depression	177	0.651 (0.472–0.898)	0.009	0.377 (0.271–0.523)	<0.001
T0	Anxiety	177	0.766 (0.564–1.040)	0.087	0.368 (0.265–0.511)	<0.001
T0	Stress	177	0.814 (0.598–1.109)	0.193	0.366 (0.262–0.510)	<0.001
T1	Depression	180	0.725 (0.545–0.965)	0.028	0.382 (0.277–0.529)	<0.001
T1	Anxiety	180	0.733 (0.542–0.991)	0.043	0.395 (0.283–0.552)	<0.001
T1	Stress	180	0.706 (0.518–0.963)	0.028	0.407 (0.291–0.571)	<0.001

Note. Separate proportional-odds ordinal logistic regression models were fitted for each DASS-21 subscale at each survey wave. Each model included one DASS-21 subscale and PSQI global score jointly and adjusted for sex, age, and previous systematic mind–body exercise experience. ORs are expressed per 1-SD higher predictor using SDs from the corresponding wave-specific complete-case sample. ORs below 1 indicate lower odds of being in a higher overall quality-of-life category. P values are descriptive sensitivity results and were not treated as an additional confirmatory testing family.

Table S8. Secondary HC3 models for DASS-21 total scores

Wave	Term	β	HC3 SE	95% CI	Raw P	Standardized β	Adjusted R²
T0	Intercept	−28.654	27.930	−83.786 to 26.478	0.306		0.155
T0	PSQI global	1.128	0.367	0.404–1.852	0.002	0.285	0.155
T0	Global QoL	−1.570	0.843	−3.234 to 0.093	0.064	−0.153	0.155
T0	Female sex	−0.596	2.201	−4.940 to 3.749	0.787	−0.023	0.155
T0	Age	2.018	1.349	−0.645 to 4.681	0.137	0.128	0.155
T0	Previous mind–body practice	0.702	1.476	−2.211 to 3.616	0.635	0.034	0.155
T1	Intercept	−3.079	21.784	−46.074 to 39.916	0.888		0.168
T1	PSQI global	1.062	0.290	0.490–1.634	<0.001	0.298	0.168
T1	Global QoL	−1.873	0.763	−3.379 to −0.367	0.015	−0.201	0.168
T1	Female sex	0.183	1.828	−3.425 to 3.792	0.920	0.007	0.168
T1	Age	0.656	1.077	−1.469 to 2.781	0.543	0.041	0.168
T1	Previous mind–body practice	0.632	1.372	−2.077 to 3.340	0.646	0.030	0.168

Note. DASS-21 total score is the continuous outcome. Models include PSQI global score, global quality of life, sex, age, and previous systematic mind–body practice. HC3 heteroscedasticity-consistent standard errors are reported. This analysis is secondary and concurrent; coefficients do not establish temporal ordering or causal effects.

Abbreviations

CI, confidence interval; DASS-21, Depression Anxiety Stress Scales – 21; ERQ, Emotion Regulation Questionnaire; HC3, heteroscedasticity-consistent covariance estimator 3; IQR, interquartile range; MAIA-2, Multidimensional Assessment of Interoceptive Awareness, Version 2; OR, odds ratio; PSQI, Pittsburgh Sleep Quality Index; QC, quality control; QoL, quality of life; SD, standard deviation; SMD, standardized mean difference.
